# Phosphoproteomic Analysis Reveals Impairment of Rice Germination by Chloramphenicol

**DOI:** 10.3390/plants14182845

**Published:** 2025-09-12

**Authors:** Rui Li, Narumon Phaonakrop, Sittiruk Roytrakul, Karan Lohmaneeratana, Arinthip Thamchaipenet

**Affiliations:** 1 Interdisciplinary Graduate Program in Bioscience, Faculty of Science, Kasetsart University, Bangkok 10900, Thailand; rui.l@ku.th; 2 Department of Genetics, Faculty of Science, Kasetsart University, Bangkok 10900, Thailand; karan.l@ku.th; 3 National Center for Genetic Engineering and Biotechnology, National Science and Technology Development Agency, Pathum Thani 12120, Thailand; narumon.pha@biotec.or.th (N.P.); sittiruk@biotec.or.th (S.R.); 4 Omics Center for Agriculture, Bioresources, Food, and Health, Kasetsart University (OmiKU), Bangkok 10900, Thailand

**Keywords:** chloramphenicol, rice (*Oryza sativa* L.) germination, phosphoproteomics, signaling pathways

## Abstract

Seed germination is a critical phase in rice production and is highly sensitive to environmental and chemical stresses. Chloramphenicol (CAM), a known phytotoxic antibiotic, has been reported to suppress rice seedling establishment, yet its underlying molecular mechanisms remain poorly understood. In this study, we investigated the effects of varying CAM concentrations on rice germination and early seedling establishment. While CAM significantly retarded germination speed and seedling growth, the final germination rates remained largely unaffected, even at high concentrations. To uncover the molecular basis of CAM phytotoxicity, we conducted time-resolved phosphoproteomic profiling during both the germination and early seedling stages. Our analyses revealed dynamic, stage-specific phosphorylation changes: moderate alterations affecting metabolic and cytokinesis-related processes during germination, and extensive disruptions in metabolic pathways, stress response mechanisms, DNA replication, and hormone signaling during early seedling establishment. Collectively, these findings demonstrate that CAM disrupts rice development by remodeling phosphorylation networks and modulating key physiological and signaling pathways. This study provides novel insights into the molecular mechanisms underlying antibiotic-induced growth inhibition and advances our understanding of plant stress responses during early development.

## 1. Introduction

Rice is a staple food for a significant proportion of the global population and plays a vital role in ensuring food security and economic stability worldwide [1]. With rising demand [2], improving rice yield remains a critical objective for production. Thailand is a major contributor to the global rice trade [3], with Khao Dawk Mali 105 (*Oryza sativa* L. cv. KDML 105), commonly known as Thai Jasmine rice, being one of its key export cultivars [4]. Although KDML 105 is favored in the market for its exceptional quality and distinctive aroma, its yield per hectare is typically lower than that of other white rice varieties [5]. Therefore, enhancing the yield of KDML 105 is crucial for bolstering Thailand’s agricultural economy and supporting the country’s contribution to the global rice trade.

Germination is the initial step in rice production, crucial for subsequent vegetative growth and ultimately influencing optimal yield. From an omics perspective, this process can be divided into two main stages: (i) the germination stage, occurring from 0 to 72 h, and (ii) the early seedling establishment stage, occurring from 72 to 168 h [6,7]. During the germination stage, seeds undergo three distinct phases: Phase I involves water uptake; Phase II entails activation of seed storage enzymes; and Phase III marks the initiation of key metabolic pathways [8]. These events culminate in the mobilization of storage reserves to support seedling establishment. Disruption of this process can significantly compromise plant vigor and productivity, making it a key target for crop improvement efforts.

Chloramphenicol (CAM) is a broad-spectrum antibiotic that inhibits protein synthesis by binding to the 50S ribosomal subunit, thereby preventing peptide bond formation [9]. Although primarily effective against prokaryotes, CAM persists in agricultural environments through wastewater contamination [10] and soil microbial activity [11], raising concerns about its ecological impact. Previous studies have revealed that CAM exerts phytotoxic effects across different plant species [12] by disrupting key physiological processes such as nutrient and water uptake [13,14], photosynthesis [15], reactive oxygen species (ROS) homeostasis [15]. These disruptions underscore CAM’s role as a potential environmental stressor in agroecosystems. In the context of seed biology, CAM has been proposed to inhibit germination by suppressing α-amylase activity [16] and compromising membrane integrity through hydrophobic interactions [17]. In tomato, CAM was found to inhibit early seedling establishment more strongly than germination initiation [18], suggesting stage-specific sensitivity.

Plant hormones regulate seed germination and early seedling growth in response to environmental cues. Early work by Noodén and Thimann (1965) demonstrated that CAM significantly disrupts auxin-mediated plant growth during germination. They further showed that exogenous application of indole-3-acetic acid (IAA) partially alleviates CAM-induced growth inhibition [19], suggesting that CAM interferes with the auxin signaling pathway. Although it was proposed that this interference results from the inhibition of protein biosynthesis required for auxin signaling [19], the specific protein targets affected by CAM remain unidentified. Given CAM’s established role as a protein biosynthesis inhibitor, investigating CAM-induced proteomic alterations is critical for elucidating its underlying phytotoxic mechanisms.

Protein phosphorylation is a critical post-translational modification that regulates protein structure and function, playing essential roles in enzyme activity, signal transduction, and stress responses [20]. Phosphoproteomic analysis enables dynamic, system-level profiling of protein activation states and underlying signaling networks. Prior studies have demonstrated that phosphorylation patterns in rice seeds change dynamically during germination. Notably, proteins involved in hormonal signaling are predominantly phosphorylated during early germination, while those linked to storage mobilization and stress adaptation dominate later stages [21]. Additionally, spatial phosphoproteomic analyses have revealed phosphorylation of nuclear zinc finger proteins as regulators of germination at the subcellular level [22]. These findings highlight phosphoproteomics as a promising approach to elucidate how rice responds to CAM-induced stress during germination.

To address current knowledge gaps, the present study employed time-resolved phosphoproteomic analysis using LC-MS/MS to characterize dynamic changes in protein phosphorylation across distinct germination stages under CAM treatment. By identifying key regulatory proteins and signaling pathways affected by CAM, this work provides novel insights into the molecular basis of antibiotic-induced phytotoxicity and lays the groundwork for developing strategies to mitigate its adverse effects on rice productivity.

## 2. Results

### 2.1. The Effect of Chloramphenicol (CAM) on Seed Germination

**Figure 1:**
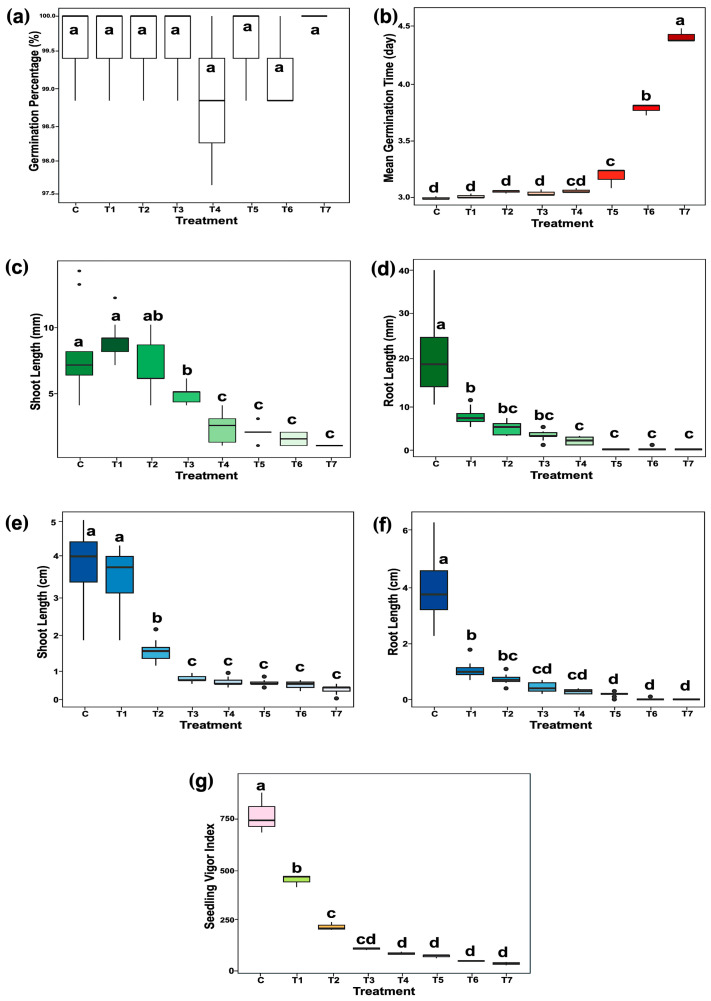
Effects of chloramphenicol (CAM) in rice (*Oryza sativa* L. cv. KDML 105) at 3-day (germination stage) and 6-day (early seedling establishment stage). (**a**) Germination percentage, (**b**) median germination time, (**c**) shoot length at 3-day, (**d**) shoot length at 6-day, (**e**) root length at 3-day, (**f**) root length at 6-day, and (**g**) seedling vigor index (SVI). Different lowercase letters above the bars indicate significant differences between treatments based on Tukey’s HSD test (*p* < 0.05). C, water control; T1, T2, T3, T4, T5, T6, and T7 represent treatments of 5, 15, 50, 150, 500, 1500, and 2500 µg/mL CAM, respectively.

The investigation of the effects of CAM (FUJIFILM Wako Pure Chemical Corporation, Osaka, Japan) at various concentrations suggests that CAM minimally affects overall seed germinability. No significant differences in germination rates were observed between the CAM-treated seeds and the water control, with all groups achieving germination rates above 98% (Figure 1a). Furthermore, treatments with 500 µg/mL, 1500 µg/mL, and 2500 µg/mL CAM significantly delayed the median germination time from 3 to 4.5 days, whereas no significant differences were observed between the control and the lower concentration treatments (Figure 1b). In contrast to its minimal effect on germination, CAM treatment significantly inhibited seedling establishment. Exposure to CAM concentrations above 15 µg/mL led to a noticeable reduction in shoot length at 3-day (germination stage) (Figure 1c). This inhibitory effect became more pronounced at 6-day (early seedling establishment stage), resulting in substantial suppression of shoot growth (Figure 1d). A similar result was observed in root development. Root growth was strikingly restricted when exposed to CAM concentrations above 5 µg/mL during the germination stage and was markedly reduced during the early seedling establishment stage (Figure 1e,f). The seedling vigor index (SVI), which integrates germination percentage and seedling growth, was used to assess overall seedling performance under CAM treatment. A significant reduction in SVI was observed even at the lowest CAM concentration of 5 µg/mL. However, increasing the CAM concentration beyond 50 µg/mL did not result in further statistically significant changes in SVI (Figure 1g).

**Figure 2:**
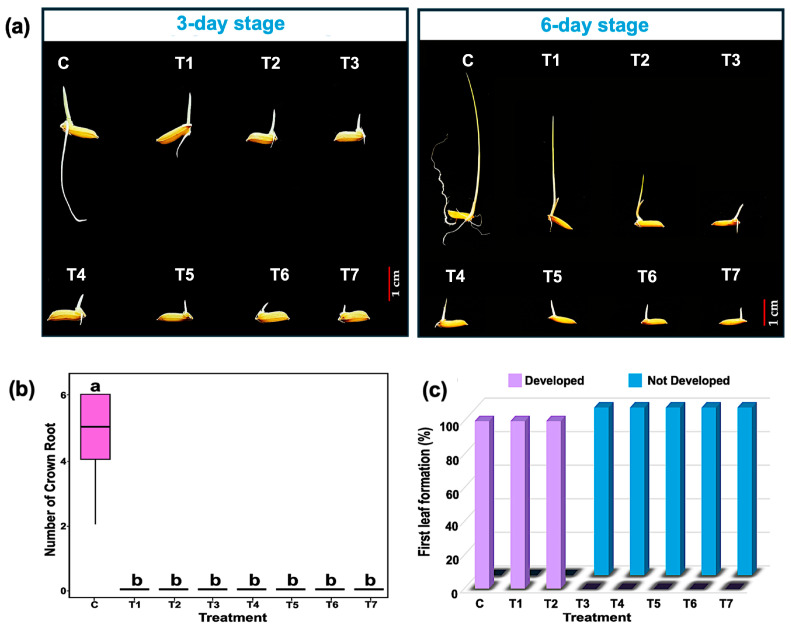
Effects of chloramphenicol (CAM) on the morphology of rice (*Oryza sativa* L. cv. KDML 105) seedlings. (**a**) Representative seedling phenotypes at 3-day (germination stage) and 6-day (early seedling establishment stage), (**b**) crown root number, and (**c**) percentage of first leaf formation. Different lowercase letters above the bars indicate significant differences between treatments based on Tukey’s HSD test (*p* < 0.05). C, water control; T1, T2, T3, T4, T5, T6, and T7 represent treatments of 5, 15, 50, 150, 500, 1500, and 2500 µg/mL CAM, respectively.

Moreover, CAM exposure caused marked alterations in rice seedling morphology (Figure 2a). Crown root formation was completely inhibited at all tested CAM concentrations (Figure 2b). No normal seedlings, with both root and first leaf, developed when seeds were treated with CAM concentrations exceeding 15 µg/mL (Figure 2c). While no statistically significant differences in SVI were observed between the 15 and 50 µg/mL CAM treatments (Figure 1g), 15 µg/mL was the highest concentration that allowed for the successful establishment of normal seedlings. Based on this, 15 µg/mL CAM was selected for sample preparation in the subsequent phosphoproteomic analysis.

### 2.2. The Effect of CAM on the Phosphoprotein Profiles of Germinating Seeds

**Figure 3:**
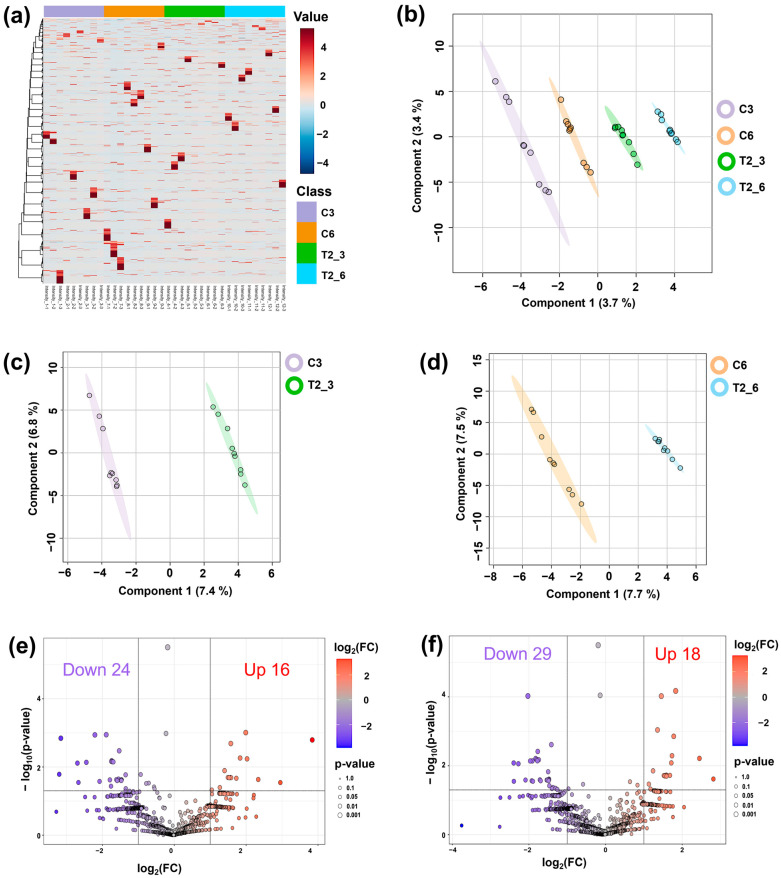
Phosphoprotein profiles of germinated rice seeds (*Oryza sativa* L. cv. KDML 105) in response to CAM at 3-day (germination stage) and 6-day (early seedling establishment stage). (**a**) Heatmap depicting the phosphoprotein profiles of CAM-treated rice at 3-day and 6-day. Two-dimensional PLS-DA plots of phosphoprotein profiles of (**b**) controls and CAM-treated rice at 3-day and 6-day, (**c**) control and CAM-treated rice at 3-day, (**d**) control and CAM-treated rice at 6-day. Volcano plots of CAM-responsive phosphoproteins at (**e**) 3-day and (**f**) 6-day. C3, 3-day control; C6, 6-day control; T2_3, 3-day CAM treatment; and T2_6, 6-day CAM treatment.

Phosphoprotein samples derived from germinated seeds treated with sterile distilled water (control) and 15 µg/mL CAM (treatment) were analyzed using LC-MS/MS. A total of 8509 phosphoproteins were identified (Table S1). This analysis revealed that CAM treatment altered the global phosphoprotein profiles of germinating seeds (Figure 3a). Partial least squares discriminant analysis (PLS-DA) further resolved the data into four distinct clusters, demonstrating statistically significant variations between control and CAM treatment across the two germination stages (Figure 3b). Stage-specific PLS-DA analyses produced comparable results (Figure 3c,d). Subsequently, the differentially expressed phosphoprotein (DEPP) analysis revealed that at the germination stage (3-day), 16 phosphoproteins were upregulated, while 24 were downregulated (Figure 3e, Table S2). At the early seedling establishment stage (6-day), 18 phosphoproteins were upregulated, and 29 were downregulated (Figure 3f, Table S2). These DEPPs were identified as CAM-responsive phosphoproteins for further bioinformatics analysis.

**Figure 4:**
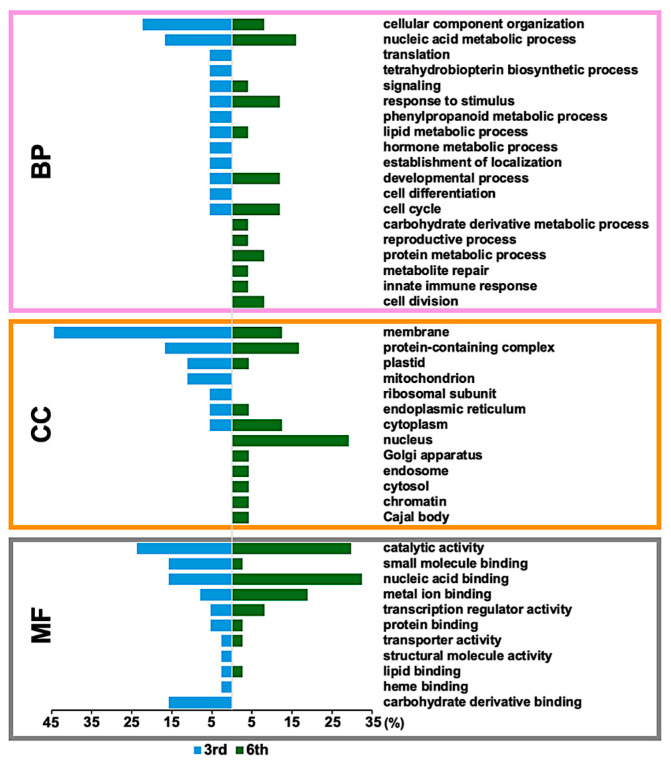
Gene Ontology (GO) annotation of identified CAM-responsive phosphoproteins at 3-day (germination stage) and 6-day (early seedling establishment stage). MF, molecular function; BP, biological process; CC, cellular component; blue bar, 3-day; and green bars, 6-day annotations.

Gene ontology (GO) annotation indicated that CAM-response phosphoproteins across a broad spectrum of biological processes (Figure 4). Several biological processes, including cellular component organization, nucleic acid metabolism, response to stimulus, development, cell cycle, signaling, and lipid metabolism, were influenced by CAM independent of developmental stage. Biological processes related to tetrahydrobiopterin, hormone metabolism, and cell differentiation were specifically activated at the germination stage (3-day), whereas biological processes associated with carbohydrate derivative metabolism, protein metabolism, cell division, and the innate immune response became more prominent at the early seedling establishment stage (6-day). Similarly, CAM treatment affected a variety of cellular components, with an observable shift from membrane, plastid, and mitochondrion localization at the germination stage to nucleus, cytoplasm, Golgi apparatus, endosome, and Cajal body localization at 6-day. Catalytic activity, metal ion binding, and nucleic acid binding were the top three molecular functions associated with the identified phosphoproteins at both stages (Figure 4).

### 2.3. Enrichment Analysis of Identified CAM-Responsive Phosphoproteins

**Figure 5:**
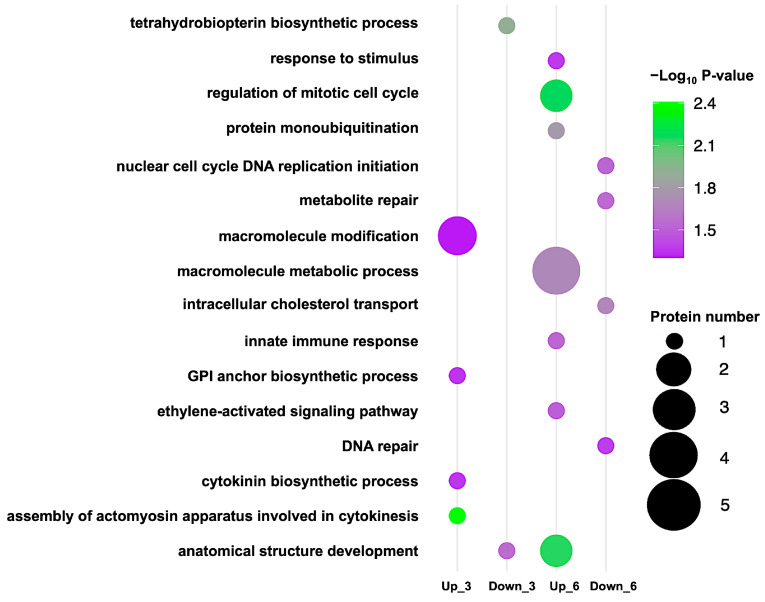
Bubble plot of pathway enrichment in CAM-response phosphoproteins at 3-day (germination stage) and 6-day (early seedling establishment stage). Bubble corresponds to a biological pathway, bubble size indicates the number of enriched proteins, and bubble color reflects the direction of regulation. The x-axis shows different conditions of upregulated CAM-response phosphoproteins at 3-day (Up_3) and 6-day (Up_6) and downregulated CAM-response phosphoproteins at 3-day (Down_3) and 6-day (Down_6). The y-axis categorizes pathways by their annotations.

Pathway enrichment analysis revealed that CAM’s impact on germinating rice seeds intensified over time (Figure 5). At 3-day (germination stage), CAM upregulated phosphoproteins involved in macromolecule modification, GPI-anchor biosynthesis, cytokinin biosynthesis, and cytoskeleton-dependent cytokinesis, while downregulating those associated with tetrahydrobiopterin biosynthesis and anatomical structure development. By 6-day (early seedling establishment stage), CAM-responsive phosphoproteins were predominantly enriched in pathways related to macromolecule metabolism, regulation of the mitotic cell cycle, anatomical structure development, response to stimuli, protein monoubiquitination, the innate immune response, and ethylene-activated signaling. In contrast, proteins involved in metabolite repair, intracellular cholesterol transport, DNA replication, and DNA repair were downregulated. Notably, phosphoproteins related to anatomical structure development responded to CAM at both stages, with greater enrichment observed at the later stage (Table 1).

## 3. Discussion

### 3.1. CAM-Induced Physiological and Phosphoproteomic Changes During Germination

The physiological results demonstrated that although a high concentration of chloramphenicol (CAM) (2500 µg/mL, approaching its solubility limit in water) significantly delayed median germination time from 3 to 4.5 days, the germination rate was still reasonably high (98%, Figure 1a,b). This observation aligns with previous research in lettuce, which reported that 3000 µg/mL CAM had minimal effects on seed germinability [23].

In contrast, all tested CAM concentrations significantly inhibited seedling growth, with the degree of inhibition varying by organ and developmental stage. Roots—highly responsive to environmental stimuli during rice germination [24]—were more sensitive to CAM than shoots. Primary root growth was markedly suppressed beginning at 5 µg/mL CAM during both germination and early seedling establishment, whereas shoot growth remained unaffected at this concentration (Figure 1e,f). This organ-specific sensitivity concurs with previous studies in *Poaceae* and *Brassicaceae* reporting similar root inhibition at comparable CAM levels [12], likely due to preferential CAM accumulation in root tissues as observed in broad bean plants [25]. Additionally, CAM suppressed crown root formation during germination (Figure 2a,b). These results suggest that the reduction in seedling vigor is not solely a consequence of delayed germination but also reflects broader CAM-induced toxicity affecting seedling physiology.

At the molecular level, CAM-induced alterations in phosphorylation profiles exhibited clear stage specificity. Among the identified differentially expressed phosphoproteins (DEPPs), only two—A0A0P0X308 and Q2QQ28—were consistently downregulated across both germination stages (Figure S1 and Table S6). A0A0P0X308 remains uncharacterized, whereas Q2QQ28 encodes a zinc finger protein of the Cys-Cys-His-Cys (CCHC) type, which is predicted to localize to the nucleus (Figure S2). Notably, previous studies have shown that altering the stage-specific phosphorylation of nucleus-localized zinc finger proteins can significantly delay rice germination [22]. Therefore, our findings suggest that CAM may slow rice germination by modifying the phosphorylation status of this CCHC-type zinc finger protein.

Further enrichment analysis revealed clear stage-specific differences (Figure 5) that support our physiological observations. At the early seedling establishment stage, CAM-responsive phosphoproteins participated in a broader spectrum of biological processes compared to those observed at the germination stage. This finding aligns with previous reports describing dynamic phosphoproteomic changes during early rice germination [26] and supports observations of the accelerated inhibitory effects of CAM on plant growth [19]. Notably, a significant increase in the number of nucleus-localized CAM-responsive phosphoproteins was detected at the later germination stage (Figure 4, Table 1), diverging from earlier studies that documented a general decline in nuclear phosphoproteins during germination [22]. Nevertheless, CAM-responsive phosphoproteins associated with morphological development were identified at both stages, underscoring the impact of CAM on developmental regulation during rice germination.

Collectively, these physiological and phosphoproteomic results reveal that sensitivity to CAM during germination is both organ-specific and dependent on the developmental stage. Furthermore, the effects of CAM become more pronounced over time, progressively affecting a broader and more complex array of biological processes as the duration of exposure extends.

### 3.2. The Impact of CAM Exposure on the 3-Day Germination Stage in Rice

#### 3.2.1. CAM Treatment Disrupts Phragmoplast-Based Cytokinesis

**Figure 6:**
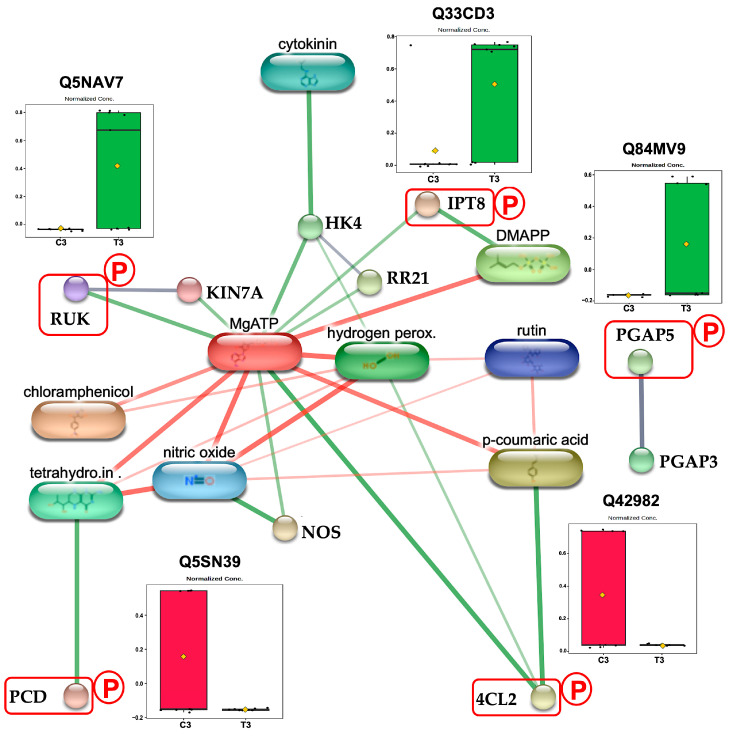
Protein–protein interaction network of CAM-responsive phosphoproteins and their potential functional partners identified at the germination stage (3-day). Phosphoproteins identified in this study are highlighted with a red square marked ‘P’, and the abundance of each protein under control (C) and treatment (T) conditions is displayed in a box plot with statistical comparisons using Fisher’s LSD test (*p* < 0.05). Red and green boxes represent phosphoproteins abundance in control and treatment samples, respectively.

RUNKEL (RUK) (Q5NAV7), a member of the FUSED serine/threonine kinase family, regulates phragmoplast-based cytokinesis by directly binding and modulating the localization of microtubule-associated proteins (MAPs) [27], including the kinesin-like protein KIN-7A [28], a key promoter of phragmoplast expansion [29]. Loss of RUK function causes cytokinesis defects due to KIN-7A mislocalization [28]. In this study, exposure to CAM for 72 h significantly increased phosphorylated RUK levels in germinating rice (Figure 6). Although RUK is classified as a pseudokinase and likely lacks catalytic activity [30], its phosphorylation may interfere with its ability to bind MAP targets. Given that mislocalization of KIN-7A leads to growth inhibition [28], we propose that CAM-induced phosphorylation diminishes RUK’s binding affinity, resulting in MAP mislocalization and the subsequent disruption of phragmoplast-based cytokinesis. This hypothesis is further supported by protein network analysis, which reveals predicted interactions between RUK and KIN-7A (Figure 6).

#### 3.2.2. CAM Disrupts GPI Anchor Protein Maturation

The Os03g0397300 protein (Q84MV9) is a rice homolog of post-GPI attachment to proteins factor 5 (PGAP5). In eukaryotes, PGAP5 functions primarily as a metallophosphoesterase that regulates glycan core remodeling during glycosylphosphatidylinositol (GPI) anchor biosynthesis [31]. This remodeling occurs in the endoplasmic reticulum (ER) and is essential for the efficient transport of GPI-APs from the ER to the Golgi apparatus [31,32], where they undergo further maturation via PGAP3-mediated lipid remodeling before being delivered to the plasma membrane and cell wall [33,34]. In plants, GPI anchor–mediated protein localization is important for cell wall formation and seed germination [35]. Disruption of lipid remodeling during GPI anchor biosynthesis reduces cellulose production, consequently inhibits growth in rice [36]. In this study, we found that three days of CAM exposure increases PGAP5 phosphorylation in rice (Figure 6). Although direct evidence for phosphorylation’s role in PGAP5 regulation is lacking, the growth inhibition observed under CAM treatment—consistent with that seen in *PGAP5* mutants [37]—suggests that CAM-induced phosphorylation may interfere with PGAP5 activity. We propose that CAM suppresses seedling growth by promoting PGAP5 phosphorylation, which disrupts glycan core remodeling, reduces transport of GPI-anchored proteins from the ER to the Golgi, and ultimately weakens cell wall formation.

#### 3.2.3. CAM Treatment Enhances Mitochondrial CK Biosynthesis

Adenylate isopentenyltransferase 8 (IPT8, Q33CD3) regulates cytokinin (CK) biosynthesis primarily by catalyzing the transfer of an isopentenyl group from dimethylallyl pyrophosphate (DMAPP) to adenine nucleotides, mainly AMP [38,39]. IPT8 expression is predominantly localized in roots and positively correlates with endogenous trans-zeatin levels, the major active CK form in plants [40,41]. Overexpression of AtIPT8 has been shown to impair seedling growth, particularly root development, by increasing CK levels, which in turn elevate reactive oxygen species (ROS) such as hydrogen peroxide (H_2_O_2_) [42]. Although CK signaling in rice involves a well-characterized phosphorelay cascade—including the histidine kinase receptor OsHK4 and the type-B response regulator OsRR21 [43,44]—the functional consequences of OsIPT8 phosphorylation on its enzymatic activity remain unknown. In this study, we observed increased phosphorylation of OsIPT8 following 72 h of CAM treatment (Figure 6). Given that the morphological changes induced by CAM resemble the AtIPT8 overexpression phenotype [42], we hypothesize that CAM-induced phosphorylation activates OsIPT8, thereby enhancing cytokinin biosynthesis and contributing to growth inhibition through elevated oxidative stress.

#### 3.2.4. CAM Exposure Disrupts Nitric Oxide Biosynthesis

Pterin-4-alpha-carbinolamine dehydratase (PCD) is a key enzyme in tetrahydrobiopterin (BH_4_) metabolism, catalyzing the conversion of 4a-hydroxytetrahydrobiopterin back into its active form [45,46]. Given that BH_4_ serves as an essential cofactor for nitric oxide synthase (NOS) [47], PCD indirectly regulates nitric oxide (NO) production [48]. In rice, inhibition of NOS-mediated NO synthesis has been shown to compromise oxidative stress tolerance, resulting in impaired germination and root development [49,50]. In our study, we detected a decrease in phosphorylated PCD (Q5SN39) levels after 72 h of CAM treatment (Figure 6). While direct evidence linking phosphorylation to PCD activation remains to be established, the well-documented role of NO in rice stress tolerance [51] supports the hypothesis that CAM-induced reduction in PCD phosphorylation disrupts BH_4_ metabolism and NO signaling, thereby contributing to the observed germination and growth deficits.

#### 3.2.5. CAM Treatment Reduces Flavonoid Metabolic Pathway

4-Coumarate-CoA ligase 2 (4CL2) catalyzes the conversion of 4-coumaric acid into 4-coumaroyl-CoA, a key intermediate in the flavonoid biosynthesis pathway [52]. Unlike other members of the 4CL family, 4CL2 is primarily associated with flavonoid production rather than lignin formation [53]. In *Camellia sinensis*, the expression level of Cs4CL2 has been shown to correlate with the endogenous accumulation of quercetin-3-O-rutinoside (rutin) [54], a major flavonoid compound. Rutin contributes to plant development through its role in anatomical structure formation, and exogenous application of rutin has been reported to promote plant growth [55]. Moreover, rutin functions as a potent antioxidant [56], enhancing stress tolerance by facilitating the scavenging of ROS [57], including hydrogen peroxide (H_2_O_2_) [58]. In rice, Os4CL2 displays high enzymatic activity in roots [53], and increased accumulation of rutin under salinity stress has been identified as part of the adaptive response to abiotic stress conditions [59]. Although serine residues have been identified as potential phosphorylation sites in Bn4CL3 [60], the regulatory role of phosphorylation in 4CL enzymes remains largely uncharacterized. In this study, we observed that CAM treatment significantly reduced the phosphorylation level of Os4CL2 (Q42982) (Figure 6) as well as root development during early germination (3-day). Given the established role of rutin in promoting rice root growth [61], our findings suggest that phosphorylation may be critical for Os4CL2 activation and subsequent rutin biosynthesis. We propose that CAM-mediated dephosphorylation diminishes Os4CL2 activity, leading to reduced rutin production and impaired ROS scavenging capacity, thereby negatively affecting root development.

### 3.3. The Impact of CAM Exposure on the 6-Day Early Seedling Establishment Stage in Rice

#### 3.3.1. CAM Enhances Nuclear Export of SHR

**Figure 7:**
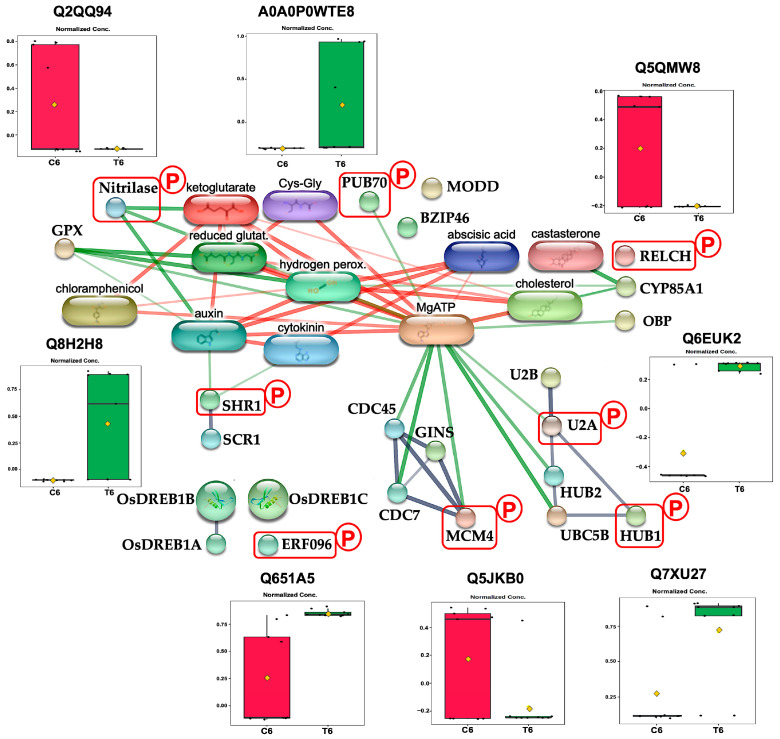
Protein–protein interaction network of CAM-responsive phosphoproteins and their predicted functional partners at the early seedling establishment stage (6-day). Phosphoproteins identified in this study are highlighted with a red square marked ‘P’, and the abundance of each protein under control (C) and treatment (T) conditions is displayed in a box plot with statistical comparisons using Fisher’s LSD test (*p* < 0.05). Red and green boxes represent phosphoproteins abundance in control and treatment samples, respectively.

SHORT-ROOT (SHR) is a pivotal transcription factor specifically expressed in stele cells that regulates plant growth, particularly root architecture, by coordinating cell division and tissue patterning [62,63]. For SHR to function as a non-cell-autonomous regulator of root cell differentiation, it must be exported from the nucleus into the cytoplasm of stele cells, allowing its movement via plasmodesmata into adjacent endodermal cells [64]. Within endodermal cells, SHR interacts with SCARECROW (SCR) and accumulates in the nucleus to regulate gene expression [65]. In rice, OsSHR1 similarly regulated root development through interactions with SCR in the endodermis [66], and loss-of-function mutants of OsSHR1 inhibited seedling growth and impaired root development [67]. In *Arabidopsis*, SHR phosphorylation at specific threonine residues modulates its nuclear export and intercellular trafficking [64], suggesting post-translational modification as a key regulatory mechanism. Computational predictions from our study indicate a high potential for the serine phosphorylation of OsSHR1 in rice under CAM treatment (Figure S3). Consistent with this, we observed a significant upregulation of phosphorylated OsSHR1(Q8H2X8) levels after 144 h of CAM treatment (Figure 7). Intriguingly, the phenotypic effects of CAM treatment resemble those observed in OsSHR1 loss-of-function mutants, characterized by reduced root development [67]. These findings suggest that CAM-induced phosphorylation of OsSHR1 may impair its mobility between cells, consequently disrupting its regulatory functions and reducing cell division during rice germination.

#### 3.3.2. CAM Exposure Distributs Histone Modification

Histone monoubiquitination 1 (HUB1) functions as an E3 ubiquitin ligase, collaborating with HUB2 and E2 ubiquitin-conjugating enzymes (UBCs) to catalyze the monoubiquitination of histone H2B (H2Bub) by attaching a single ubiquitin molecule [68]. This histone modification is a critical regulatory mechanism essential for rice development and stress adaptation [69,70]. Disruption of HUB1 expression impairs rice growth [70]. In the present study, we observed that phosphorylated OsHUB1 (Q7XU27) levels were significantly elevated after 144 h of CAM treatment (Figure 7). Notably, this increase in OsHUB1 phosphorylation was accompanied by reduced seedling growth, which contrasts with earlier reports indicating that HUB1-mediated H2Bub promotes plant growth [71,72]. This inconsistency may be due to the regulatory effects of phosphorylation on E3 ubiquitin ligase activity. For instance, in *Arabidopsis*, phosphorylation by SnRK2 kinases suppresses the E3 ligase function of HUB2 without disrupting the HUB1/HUB2 complex [73]. Based on these findings together with our results, we propose that CAM treatment induces phosphorylation that inactivates HUB1’s E3 ligase activity, thereby reducing H2Bub levels and inhibiting seedling growth.

#### 3.3.3. CAM Treatment Suppresses AP2/ERF-Mediated Stress Tolerance

The Os09g0571700 protein (Q651A5), an ethylene-responsive transcription factor 96 (ERF096), is a DNA-binding protein that specifically targets the GCC-box and dehydration-responsive element (DRE) cis-elements [74]. In rice, OsERF096 functions as a negative regulator of stress tolerance by repressing dehydration-responsive element-binding (DREB) factors [74], which are key transcription factors that activate stress-responsive genes through an ABA-independent signaling pathway [75,76]. We found that CAM treatment significantly increased the levels of phosphorylated OsERF096 during the early seedling establishment stage (6-day) (Figure 7). Although phosphorylation is known to be essential for OsERF activation [77,78,79], direct evidence of its role in regulating OsERF096 remains lacking. Given the consistent growth inhibition observed in OsERF096 overexpression mutants [74], our results suggest that CAM-induced phosphorylation may be necessary for OsERF096 activation. Taken together, the CAM-induced increase in phosphorylated OsERF096 appears to enhance its repressive effect on the expression of OsDREB1 genes, thereby reducing rice growth under CAM treatment conditions.

#### 3.3.4. CAM Exposure Influence Pre-mRNA Splicing

U2 small nuclear ribonucleoprotein A (U2A) is a core component of the U2 small nuclear ribonucleoprotein (U2 snRNP), which is essential for the function of the major spliceosome [80]. U2A contributes to pre-mRNA splicing by forming a heterodimer with U2B [81]. Disruption of this complex results in severe splicing defects [81], which can compromise cell wall integrity and reduce stress tolerance [82]. In plants, U2A is both evolutionarily conserved and stress-responsive [80], with its expression upregulated under stress conditions [83]. Although phosphorylation is a well-established regulatory mechanism in U2 snRNP-mediated splicing [84], the specific functional consequences of U2A phosphorylation remain unclear. Our proteomic analysis revealed elevated levels of U2A (Q6EUK2) phosphorylation after 144 h CAM treatment (Figure 7). Given the observed phenotypes, which are consistent with splicing defects [85], we propose that CAM-induced phosphorylation of U2A disrupts its heterodimerization with U2B, thereby impairing U2 snRNP-mediated splicing. This disruption likely compromises cell wall integrity and ultimately inhibits seedling growth.

#### 3.3.5. CAM Exposure Reduce ABA-Mediated Stress Response

Os06g0163000 protein (A0A0P0WTE8), a plant U-box protein 70 (PUB70), functions as a U-box E3 ubiquitin ligase. It negatively regulates abscisic acid (ABA) signaling by mediating the ubiquitination and subsequent degradation of OsbZIP46 through its interaction with the mediator of OsbZIP46 deactivation and degradation (MODD) [86]. The E3 ubiquitin ligase activity of OsPUB70 is essential for OsbZIP46 ubiquitination and degradation [86]. Loss of OsPUB70 function impairs the degradation of OsbZIP46, which in turn leads to increased ABA sensitivity [86]. Our proteomic analysis shows that phosphorylated OsPUB70 levels increase during the early seedling establishment stage (Figure 7) in response to CAM treatment. Given the demonstrated disruption of protein phosphorylation on the activation of E3 ubiquitin ligases [87], we propose that increased phosphorylation induced by CAM may inactivate the E3 ubiquitin ligase activity of OsPUB70, thereby reducing the degradation of OsbZIP46. This, in turn, could ultimately inhibit growth by enhancing ABA sensitivity in rice.

#### 3.3.6. CAM Exposure Impairs Castasterone Biosynthesis

The HEAT repeat-containing protein Os01g0514300 (Q5QMW8) belongs to the RELCH family, which facilitates cholesterol non-vesicular transport between recycling endosomes and the trans-Golgi network through interactions with the small GTPase RAB11 and oxysterol-binding protein (OBP) [88,89]. In plants, non-vesicular transport is the primary mechanism for cholesterol trafficking and is essential for maintaining cholesterol homeostasis [90]. This pathway efficiently delivers cholesterol to biosynthetic enzymes, such as cytochrome P450 85A1 (CYP85A1), supporting the biosynthesis of castasterone (CAS) [91]. Disruption of cholesterol-derived CAS biosynthesis has been shown to impair seedling development [91]. In rice, CAS is the predominant biologically active brassinosteroid (BR), with a more significant role than brassinolide [92]. Applying CAS externally has been shown to promote seedling growth in rice when exposed to low-temperature stress [93]. In the present study, we observed that CAM treatment reduces the phosphorylation levels of RELCH (Figure 7). Although direct evidence linking phosphorylation to RELCH activity remains to be established, the phenotypic similarity between our observation and those seen in cholesterol-driven CAS production inhibition mutants in *Arabidopsis* [91], as well as BR biosynthesis inhibition phenotypes in rice [94], suggests that phosphorylation is essential for RELCH activation in cholesterol-driven CAS production. Therefore, we propose that CAM treatment reduces RELCH phosphorylation, disrupts cholesterol non-vesicular transport, and consequently suppresses seedling growth under stress by diminishing CAS production.

#### 3.3.7. CAM Exposure Increases Deaminated Glutathione

Nitrilase 1 (NIT1) functions as a metabolite repair enzyme by catalyzing the hydrolysis of deaminated glutathione (dGSH)—a toxic byproduct of glutathione metabolism—into α-ketoglutarate and cysteinylglycine [95]. This reaction prevents the accumulation of dGSH and contributes to the maintenance of the cellular antioxidant GSH pool [96]. Loss of NIT1 function causes accumulation of dGSH, which potentially inhibits glutathione peroxidase (GPX) activity [96]. This inhibition compromises the detoxification of hydrogen peroxide and lipid hydroperoxides, leading to elevated lipid peroxidation and oxidative damage to cellular membranes [97]. Our proteomic analysis revealed a significant decrease in the phosphorylation level of NIT1 homolog Q2QQ94 at the early seedling establishment stage after CAM treatment (Figure 7). Although the role of phosphorylation in regulating NIT1 remains unclear, the observed root growth inhibition—resembling the phenotype of NIT1 knockout mutants of rice? [98]—suggests that phosphorylation is necessary for the amidase activation of NIT1. We propose that CAM-induced phosphorylated NIT1 reduction leads to the accumulation of dGSH, which increases oxidative damage to cellular membranes and ultimately inhibits seedling growth.

#### 3.3.8. CAM Exposure Impairs DNA Replication

Mini-chromosome maintenance complex protein 4 (MCM4, Q5JKB0) is a vital subunit of the MCM2–7 helicase complex, which is essential for DNA replication [99]. During the G1 phase of the cell cycle, the MCM2–7 complex is loaded onto replication origins as an inactive double hexamer and is subsequently activated in the S phase [100]. This activation depends on the phosphorylation of MCM4 by cell division cycle 7 (CDC7) kinase, which facilitates the recruitment of CDC45 and the GINS complex to form the active CDC45–MCM–GINS (CMG) helicase [100]. Failure to phosphorylate MCM4 disrupts CMG assembly, impairs helicase activity, and ultimately inhibit cell proliferation by compromises DNA replication [101]. In this study, we observed a reduction in phosphorylated MCM4 levels on the sixth day of CAM treatment (Figure 7). The observed growth inhibition is consistent with the phenotype of MCM4 knockout mutants of xxx? [102], suggests that phosphorylation is essential for MCM4 activation. We propose that the CAM-induced phosphorylated MCM4 reduction disrupts CMG complex formation, thereby impairing DNA replication and inhibiting seedling establishment in rice during the early seedling establishment stage.

### 3.4. CAM Inhibits Root System Development During Germination

#### 3.4.1. CAM Inhibits Primary Root Formation at Germination Stage

**Figure 8:**
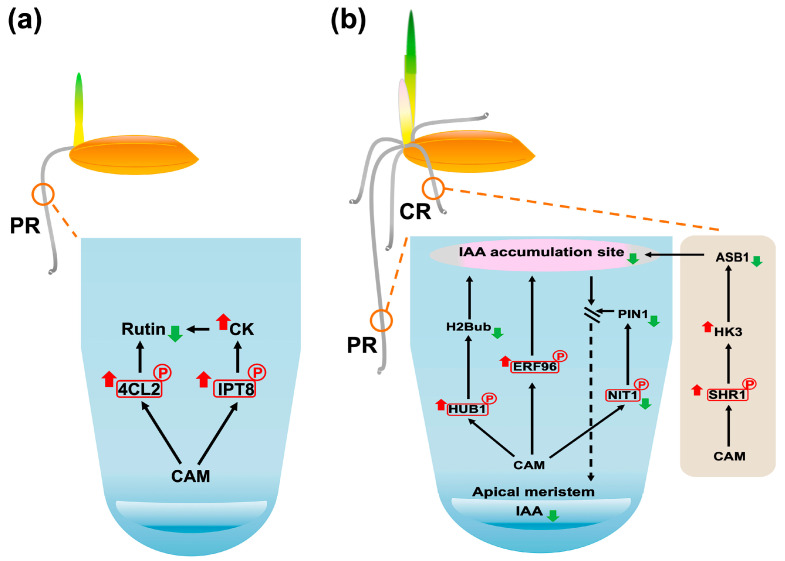
Identified phosphoproteins and potential pathways associated with root system development in rice during CAM treatment at (**a**) germination stage (3-day) and (**b**) early seedling establishment stage (6-day). PR, primary root; CR, crown root; red arrows, upregulation; green arrows, downregulation; black arrows, downstream consequences; double lines and dashed arrows, blocked IAA transport processes; and red square marked ‘P’, phosphorylated proteins.

During the 3-day germination stage, CAM treatment significantly decreased the phosphorylation level of 4CL2 (Q42982, Figure 6), a key enzyme involved in flavonoid biosynthesis, particularly affecting rutin production (Section 3.2.5). Enrichment analysis further revealed a strong association between 4CL2 and anatomical structure development (Table 1), highlighting its potential role in seedling establishment. Considering that rutin has been reported to promote root growth in rice seedlings [61], we propose that CAM suppresses primary root elongation during germination by inhibiting rutin biosynthesis. This hypothesis is supported by evidence that CK signaling negatively regulates rutin biosynthesis in *Morus alba* L. [103]. Notably, CAM treatment was correlated with enhanced CK biosynthesis, as indicated by increased phosphorylation of the CK biosynthesis enzyme IPT8 (Section 3.2.3). Taken together, these findings suggest that CAM inhibits primary root growth during the germination stage by attenuating rutin biosynthesis via elevated CK signaling (Figure 8a).

#### 3.4.2. CAM Inhibits Primary Root Formation at Early Seedling Establishment Stage

During the early seedling establishment stage (6-day), CAM exposure significantly increased phosphorylation of HUB1 (Q7XU27, Figure 7), which may lead to reduced H2Bub levels (Section 3.3.2). Considering the well-established role of H2Bub in promoting auxin-mediated primary root growth [104] and the strong correlation between HUB1 and anatomical structure development identified by enrichment analysis (Table 1), we propose that CAM impairs primary root elongation by attenuating auxin (AUX) signaling. Moreover, CAM treatment resulted in elevated levels of active OsERF096 (Section 3.3.3), decreased expression of OsNIT1 (Section 3.3.7), and reduced mobility of SHORT-ROOT1 (SHR1) (Section 3.3.1). Given that OsERF096 facilitates the conjugation and inactivation of IAA [105], OsNIT1 is critical for maintaining proper AUX distribution in roots [98], and OsSHR1 dysfunction represses root system development by inhibiting the PIN1-mediated auxin signaling pathway [67], these molecular changes further support the hypothesis that CAM inhibits primary root elongation through disruption of AUX homeostasis and signaling (Figure 8b).

#### 3.4.3. CAM Inhibits Crown Root Formation at Early Seedling Establishment Stage

In rice, root system formation during germination is a tightly regulated process that begins with primary root emergence, followed by crown root initiation and outgrowth [106]. This developmental stage relies on the finely tuned antagonistic regulatory roles of AUX and CK signaling pathways [107,108], where a lowered AUX-to-CK ratio inhibits crown root formation [108]. In *Arabidopsis*, the SHR-SCR complex modulates HISTIDINE KINASE 3 (AHK3) activity, thereby regulating CK signaling that influences AUX biosynthesis via ANTHRANILATE SYNTHASE BETA 1 (ASB1) [109]. This regulatory network enables the SHR-SCR complex to partially govern the AUX–CK balance that is essential for proper cell differentiation.

Our proteomics results revealed that CAM treatment at the early seedling establishment stage significantly increased phosphorylation of SHORT-ROOT1 (SHR1, Q8H2X8) (Figure 7), which may inhibit the formation of the SHR-SCR complex (Section 3.3.1). The phenotypic similarity in crown root development between CAM-treated rice seedlings and CK signaling enhancement mutants [108] suggests that CAM disrupts crown root development by lower the AUX-to-CK ratio. We propose that this effect may involve reduced SHR-SCR–mediated repression of the rice AHK3 homolog, OsHK3, leading to decreased OsASB1-dependent AUX biosynthesis [43]. Taken together, these findings suggest that CAM disrupts hormone homeostasis critical for crown root formation through the SHR-SCR–OsHK3–OsASB1 regulatory pathway (Figure 8b).

## 4. Materials and Methods

### 4.1. Plant Material Preparation

Healthy Thai jasmine rice (*Oryza sativa* L. cv. KDML 105) seeds obtained from the Rice Seed Division, Rice Department, Ministry of Agriculture and Cooperatives, Thailand were surface-sterilized following a modified protocol based on Md-Zali et al. [110]. Briefly, seeds were initially soaked in sterile distilled water for 40 min at room temperature with agitation at 120 rpm, followed by immersion in 1% sodium hypochlorite for 30 min and then 75% ethanol for 2 min. Sterilization was concluded with ten rinses using sterile distilled water. To assess the impact of CAM, 80 seeds were germinated in solutions containing chloramphenicol (CAM; FUJIFILM Wako Pure Chemical Corporation, Osaka, Japan) at concentrations of 5, 15, 50, 150, 500, 1500, and 2500 µg/mL in 90 × 15 mm polystyrene Petri dishes. Sterile distilled water served as the control. The highest concentration that allowed normal seedling development was identified and used for subsequent analyses. For phosphoproteomic analysis, each replicate consisted of 50 seeds germinated in 5 mL of the selected CAM concentration in 220 mL tissue culture glass jars. Control samples were prepared under the same conditions, using sterile distilled water instead of CAM. Both germination experiments were conducted in triplicate under dark conditions at 30 ± 3 °C. Samples were collected at 3 days, representing the germination stage, and at 6 days, corresponding to the early seedling establishment stage.

### 4.2. Physiological Analysis of Rice Germination

The germination status of rice seeds was assessed by counting the number of germinated seeds at 3 and 6 days after sowing. Shoot and root lengths were measured at the same time points using a ruler. Measurements were taken from ten seeds (n = 10) at 72 h and fifteen seeds (n = 15) at 144 h. The seedling vigor index (SVI) was calculated to provide a more comprehensive assessment of seedling development, using the previously described formula [111,112]: $SVI=(Germination\;percentage\times \left(shoot\;length+root\;length\right))$.

### 4.3. Total Protein Extraction

At the 3-day and 6-day germination stages, seeds were harvested and stored at −80 °C. Total protein extraction was performed by grinding the entire germinated seed (including the primary leaf, coleoptile, seed coat, endosperm, embryo, and roots) to a fine powder under liquid nitrogen using a porcelain mortar. A 150 mg aliquot of the homogenized material was transferred to a 1.5 mL centrifuge tube and vortexed with 1% SDS solution (pH 7.0) for 30 min at room temperature [113]. Following centrifugation at 12,000× *g* for 7 min at 4 °C, the supernatant was mixed with a 1:1 (*v*/*v*) solution of 20% TCA/acetone to precipitate proteins and subsequently incubated at −20 °C for 1 h [114]. The resulting protein pellets were air-dried for 3 min and then resuspended in ultrapure water. Protein concentration was determined using the Lowry assay [115].

### 4.4. Label-Free Quantitative Phosphoproteomics Analysis Using LC-MS/MS

Prior to phosphoprotein enrichment (Pierce™ Phosphoprotein Enrichment Kit, Rockford, IL, USA), protein concentrations for each sample were adjusted to 10 mg/mL, and Halt™ Phosphatase Inhibitor Cocktail (Thermo Scientific, Rockford, IL, USA) was added at 1× concentration to inhibit phosphatase activity and preserve phosphorylation. The enriched phosphoproteins were then concentrated and desalted using a 9 kDa molecular weight cut-off membrane column and gel filtration, respectively (Thermo Scientific, Rockford, IL, USA). For in-solution digestion, 5 µg of the protein sample was completely dissolved in 10 mM ammonium bicarbonate (AMBIC). Disulfide bonds were reduced with 5 mM dithiothreitol (DTT) in 10 mM AMBIC at 60 °C for 1 h, followed by alkylation of sulfhydryl groups with 15 mM iodoacetamide (IAA) in 10 mM AMBIC for 45 min at room temperature in the dark. Trypsin digestion was performed overnight at 37 °C using sequencing grade trypsin (Promega, Madison, WI, USA) at a 1:20 enzyme-to-protein ratio (50 ng/µL trypsin concentration). Prior to LC-MS/MS analysis, digested samples were dried and reconstituted in 0.1% formic acid for protonation.

Phosphopeptide separation was performed using a nano LC-MS/MS system consisting of an Ultimate 3000 LC system (Dionex, Thermo Fisher Scientific, Waltham, MA, USA) coupled to a ZenoTOF 7600 system (SCIEX, Framingham, MA, USA). For each treatment at each time point, three biological replicates were analyzed, each with three technical replicates, resulting in a total of nine LC-MS/MS injections. A 75 μm I.D. × 15 cm Acclaim PepMap RSLC C18 column (2 μm, 100 Å, nanoViper; Thermo Fisher Scientific) was used, with the column temperature maintained at 60 °C. Mobile phases consisted of 0.1% formic acid in water (solvent A) and 0.1% formic acid in 80% acetonitrile (solvent B). Phosphopeptides were eluted using a linear gradient of 5–55% solvent B over 30 min at a constant flow rate of 0.30 μL/min. The ZenoTOF 7600 system was operated in positive ion mode with the following source parameters: ion source gas 1 at 8 psi, curtain gas at 35 psi, CAD gas at 7 psi, source temperature at 200 °C, and spray voltage at 3300 V. Data-dependent acquisition (DDA) was employed, selecting the top 50 most abundant precursor ions from each survey MS1 scan for subsequent MS/MS analysis. Dynamic exclusion was enabled, with precursors excluded for 12 s after two MS/MS sampling events (dynamic collision energy enabled). MS2 spectra were acquired in the 100–1800 *m/z* range with a 50 ms accumulation time and Zeno trap activation. Collision energy parameters were as follows: 80 V declustering potential, no DP spread, and a 0 V CE spread. Time bins were summed using all channels with a 150,000 cps Zeno trap threshold. The Top 60 DDA method cycle time was set to 3.0 s.

### 4.5. Quantification and Identification of Phosphoproteins

Protein quantitation in individual samples was carried out using MaxQuant 2.4.9.0 with the Andromeda search engine [116]. The search correlated MS/MS spectra to the Uniprot *Oryza sativa* database (downloaded on 26 April 2023). The label-free quantitation was performed using MaxQuant’s standard settings, including a maximum of three missed cleavages, a mass tolerance of 0.6 dalton, trypsin as the digesting enzyme, carbamidomethylation of cysteine as a fixed modification, phosphorylation of STY, and oxidation of methionine as a variable modification. Data Availability Statement: The MS/MS raw data and analysis are available in the ProteomeXchange Consortium under the dataset PXD050963 (https://proteomecentral.proteomexchange.org/cgi/GetDataset?ID=PXD050963, accessed on 5 August 2025) and jPOST dataset JPST003006.

Visualization and statistical analyses of the phosphoproteome data, including partial least squares discriminant analysis (PLS-DA) and differential expression analysis, were conducted using MetaboAnalyst version 6.0 [117]. Peak intensities from control and treatment groups were compared separately for each sampling time point. Data quality was improved by applying a relative standard deviation (RSD) reliability filter and a median absolute deviation (MAD) variance filter. Differentially expressed protein (DEP) analysis was then performed, and a volcano plot was generated using a fold change (FC) threshold of 2.0 and a raw *p*-value threshold of 0.05. Potential phosphorylation sites within the identified DEPs were predicted using NetPhos version 3.1 (https://services.healthtech.dtu.dk/services/NetPhos-3.1/ (accessed on 19 July 2025)) [118]. Pathway enrichment analysis of DEPs was conducted using g:Profiler (stable version available at https://biit.cs.ut.ee/gprofiler/gost (accessed on 22 March 2025)) [119], restricted to the *Oryza sativa* Japonica group. A false discovery rate (FDR) of 0.05 was applied using the Benjamini–Hochberg procedure as the significance threshold. Subcellular localization of DEPs associated with biological pathways identified by enrichment analysis was predicted using DeepLoc version 2.1 (https://services.healthtech.dtu.dk/services/DeepLoc-2.1/ (accessed on 19 July 2025)) [120]. Protein–protein interaction analysis was performed using STITCH version 5.0 (http://stitch.embl.de) [121], restricting the search to the *Oryza sativa* japonica group. A minimum interaction score of 0.4 was applied, with restrictions on first-shell and second-shell interactors.

### 4.6. Statistical Analysis

Statistical analyses and graphical representations were generated using R version 4.4.1 with the RStudio interface (version 2023.06.1.524) [122]. Analysis of variance (ANOVA) was performed to assess variance within the physiological dataset, employing a significance level of α = 0.05. Post hoc multiple comparisons of means were conducted using Tukey’s Honestly Significant Difference (TukeyHSD) test at a 95% family-wise confidence level. The ‘multcompView’ and ‘ggplot2’ R packages were utilized for data visualization [123].

## 5. Conclusions

In conclusion, this study demonstrates that chloramphenicol (CAM) effectively slows rice germination and seedling establishment without markedly affecting overall germinability, exhibiting organ-specific sensitivity. Our phosphoproteomic analysis further reveals that CAM induces stage-specific phosphorylation changes disrupting key physiological and molecular pathways. Notably, during the germination stage, CAM interferes with cytokinesis, cell wall formation, and oxidative damage resistance. During early seedling establishment, CAM additionally impacts DNA replication, cell division, stress responses, and critical hormonal signaling pathways, including auxin, abscisic acid, and brassinosteroids. These findings provide important insights into the molecular mechanisms underlying the CAM-induced inhibition of seed germination and lay a foundation for developing strategies to mitigate growth suppression caused by antibiotic contamination. Future studies comparing tissue-specific responses and variations among different rice cultivars could further elucidate differential sensitivity to CAM and help identify more tolerant genotypes or targeted mitigation strategies.

## Figures and Tables

**Table 1:** Pathway enrichment analysis results of CAM-responsive phosphoproteins of germinated rice seeds (*Oryza sativa* L. cv. KDML 105) at 3-day (germination stage) and 6-day (early seedling establishment stage).

Term Name	Term ID	−log_10_ (*p*-Value)	Count	Uniprot ID	Subcellular Localization
**Upregulation at 3-day stage**					
Assembly of actomyosin apparatus involved in cytokinesis	GO:0000912	2.408	1	Q5NAV7	Cytoplasm, Nucleus
GPI anchor biosynthetic process	GO:0006506	1.355	1	Q84MV9	Endoplasmic reticulum
Cytokinin biosynthetic process	GO:0009691	1.355	1	Q33CD3	Mitochondrion
Macromolecule modification	GO:0043412	1.302	3	Q5NAV7	Nucleus
				Q84MV9	Endoplasmic reticulum
				Q33CD3	Mitochondrion
**Downregulation at 3-day stage**					
Tetrahydrobiopterin biosynthetic process	GO:0006729	1.904	1	Q5SN39	Mitochondrion
Anatomical structure development	GO:0032989	1.558	1	Q42982	Cytoplasm
**Upregulation at 6-day stage**					
Regulation of mitotic cell cycle	GO:0007346	2.161	2	Q8H2X8	Nucleus
				Q7XU27	Nucleus
Anatomical structure development	GO:0048366	2.148	2	Q8H2X8	Nucleus
				Q7XU27	Nucleus
Protein monoubiquitination	GO:0006513	1.792	1	Q7XU27	Nucleus
Macromolecule metabolic process	GO:0043170	1.696	5	Q8H2X8	Nucleus
				Q7XU27	Nucleus
				Q651A5	Nucleus
				Q6EUK2	Nucleus
				A0A0P0WTE8	Cytoplasm
Innate immune response	GO:0045087	1.532	1	Q7XU27	Nucleus
Ethylene-activated signaling pathway	GO:0009873	1.497	1	Q651A5	Nucleus
Response to stimulus	GO:0050896	1.337	1	Q7XU27	Nucleus
**Downregulation at 6-day stage**					
Intracellular cholesterol transport	GO:0032367	1.679	1	Q5QMW8	Cytoplasm
Metabolite repair	GO:0110051	1.554	1	Q2QQ94	Cytoplasm
Nuclear cell cycle DNA replication initiation	GO:1902315	1.525	1	Q5JKB0	Nucleus
DNA repair	GO:0006281	1.379	1	Q5JKB0	Nucleus

## Data Availability

The MS/MS raw data and analysis are available in the ProteomeXchange Consortium under the dataset PXD050963 (https://proteomecentral.proteomexchange.org/cgi/GetDataset?ID=PXD050963, accessed on 5 August 2025) and jPOST dataset JPST003006.

## Supplementary Materials

The following supporting information can be downloaded at https://www.mdpi.com/article/10.3390/plants14182845/s1: Table S1. Raw data of all identified phosphoproteins from germinated rice, Table S2. CAM-response phosphoproteins identified from rice seeds at day 3 (germination stage) and day 6 (early seedling establishment stage). Table S3. Enrichment analysis results of CAM-response phosphoproteins from 3-day and 6-day rice seeds, used for bubble plot visualization. Table S4. Raw data of germination. Table S5. Raw data of root/shoot lengths (3-day and 6-day), crown root number, SVI, and first leaf formation (6-day). Table S6. Venn diagram of differentially expressed phosphoproteins (DEPPs) identified at 3-day (germination stage) and 6-day (early seedling establishment stage) in response to chloramphenicol (CAM). Figure S1. Venn diagram of differentially expressed phosphoproteins (DEPPs) identified at 3-day (germination stage) and 6-day (early seedling establishment stage) in response to chloramphenicol (CAM). Figure S2. Subcellular localization prediction of the CCHC-type zinc finger protein (Q2QQ28), generated using DeepLoc version 2.1. Figure S3. Predicted phosphorylation sites of OsSHR1 (Q8H2X8), generated using NetPhos version 3.1.

## References

[1] Dominic N., Cenggoro T.W., Pardamean B. (2023). Systematic literature review: Accelerate the rice production for global food security. AIP Conf. Proc..

[2] United States Department of Agriculture. https://downloads.usda.library.cornell.edu/usda-esmis/files/3t945q76s/tx31sh59w/np195824g/wasde0725.pdf.

[3] Co H.C., Boosarawongse R. (2007). Forecasting Thailand’s rice export: Statistical techniques vs. artificial neural networks. Comput. Ind. Eng..

[4] Thai Rice Exporters Association. http://www.thairiceexporters.or.th/statistic_2025.html.

[5] Wangcharoen W., Phanchaisri C., Daengpok W., Phuttawong R., Hangsoongnern T., Phanchaisri B. (2016). Consumer acceptance test and some related properties of selected KDML 105 rice mutants. J. Food Sci. Technol..

[6] He D., Yang P. (2013). Proteomics of rice seed germination. Front. Plant Sci..

[7] Hazra A., Das S. (2024). The molecular and metabolic events behind different germination stages of rice seeds: A metabolomics perspective. JSFA Rep..

[8] Yang P., Li X., Wang X., Chen H., Chen F., Shen S. (2007). Proteomic analysis of rice (*Oryza sativa*) seeds during germination. Proteomics.

[9] Weber M.J., DeMoss J.A. (1969). Inhibition of the peptide bond synthesizing cycle by chloramphenicol. J. Bacteriol..

[10] Pan M., Wong C.K.C., Chu L.M. (2014). Distribution of antibiotics in wastewater-irrigated soils and their accumulation in vegetable crops in the Pearl River Delta, Southern China. J. Agric. Food Chem..

[11] Nguyen L.M., Nguyen N.T.T., Nguyen T.T.T., Nguyen T.T., Nguyen D.T.C., Tran T.V. (2022). Occurrence, toxicity and adsorptive removal of the chloramphenicol antibiotic in water: A review. Environ. Chem. Lett..

[12] Chen Q.Y., Wu Z.H., Liu J.L. (2011). Ecotoxicity of chloramphenicol and Hg acting on the root elongation of crops in North China. Int. J. Environ. Res..

[13] Sutcliffe J.F. (1960). New evidence for a relationship between ion absorption and protein turnover in plant cells. Nature.

[14] Önder N. (1974). Comparative studies on water uptake and water permeability of potato tissue under the effect of indoleacetic acid, chloramphenicol and actinomycin D. Physiol. Plant..

[15] Rehman A.U., Kodru S., Vass I. (2016). Chloramphenicol mediates superoxide production in photosystem II and enhances its photodamage in isolated membrane particles. Front. Plant Sci..

[16] Srivastava B.I.S., Meredith W.O.S. (1962). Mechanism of action of gibberellic acid: Inhibition of α-amylase development during germination of barley by chloramphenicol and its reversal by gibberellic acid. Can. J. Bot..

[17] Pan M., Chu L.M. (2016). Phytotoxicity of veterinary antibiotics to seed germination and root elongation of crops. Ecotoxicol. Environ. Saf..

[18] Bellino A., Lofrano G., Carotenuto M., Libralato G., Baldantoni D. (2018). Antibiotic effects on seed germination and root development of tomato (*Solanum lycopersicum* L.). Ecotoxicol. Environ. Saf..

[19] Noodén L.D., Thimann K.V. (1965). Inhibition of protein synthesis and of auxin-induced growth by chloramphenicol. Plant Physiol..

[20] Zhang W.J., Zhou Y., Zhang Y., Su Y.H., Xu T. (2023). Protein phosphorylation: A molecular switch in plant signaling. Cell Rep..

[21] Han C., Wang K., Yang P. (2014). Gel-based comparative phosphoproteomic analysis on rice embryo during germination. Plant Cell Physiol..

[22] Li M., Yin X., Sakata K., Yang P., Komatsu S. (2015). Proteomic analysis of phosphoproteins in the rice nucleus during the early stage of seed germination. J. Proteome Res..

[23] Frankland B., Smith H. (1967). Temperature and other factors affecting chloramphenicol stimulation of the germination of light-sensitive lettuce seeds. Planta.

[24] Li R., Phaonakrop N., Lohmaneeratana K., Roytrakul S., Thamchaipenet A. (2025). Phosphoproteomic insights into the regulation of root length in rice (*Oryza sativa* L. cv. KDML 105): Uncovering key events and pathways involving phosphorylated proteins. PeerJ.

[25] Pramer D. (1954). The movement of chloramphenicol and streptomycin in broad bean and tomato plants. Ann. Bot..

[26] Han C., Yang P., Sakata K., Komatsu S. (2014). Quantitative proteomics reveals the role of protein phosphorylation in rice embryos during early stages of germination. J. Proteome Res..

[27] Krupnova T., Sasabe M., Ghebreghiorghis L., Gruber C.W., Hamada T., Dehmel V., Strompen G., Stierhof Y.-D., Lukowitz W., Kemmerling B. (2009). Microtubule-associated kinase-like protein RUNKEL needed for cell plate expansion in *Arabidopsis* cytokinesis. Curr. Biol..

[28] Krupnova T., Stierhof Y.D., Hiller U., Strompen G., Müller S. (2013). The microtubule-associated kinase-like protein RUNKEL functions in somatic and syncytial cytokinesis. Plant J..

[29] Takahashi Y., Soyano T., Kosetsu K., Sasabe M., Machida Y. (2010). HINKEL kinesin, ANP MAPKKKs and MKK6/ANQ MAPKK, which phosphorylates and activates MPK4 MAPK, constitute a pathway that is required for cytokinesis in *Arabidopsis thaliana*. Plant Cell Physiol..

[30] Preuss F., Chatterjee D., Mathea S., Shrestha S., St-Germain J., Saha M., Kannan N., Raught B., Rottapel R., Knapp S. (2020). Nucleotide binding, evolutionary insights, and interaction partners of the pseudokinase Unc-51-like kinase 4. Structure.

[31] Fujita M., Maeda Y., Ra M., Yamaguchi Y., Taguchi R., Kinoshita T. (2009). GPI glycan remodeling by PGAP5 regulates transport of GPI-anchored proteins from the ER to the Golgi. Cell.

[32] Komath S.S., Fujita M., Hart G.W., Ferguson M.A., Kinoshita T., Varki A., Cummings R.D., Esko J.D., Stanley P., Hart G.W., Aebi M., Mohnen D., Kinoshita T., Packer N.H., Prestegard J.H., Schnaar R.L., Seeberger P.H. (2022). Glycosylphosphatidylinositol Anchors. Essentials of Glycobiology.

[33] Fujita M., Umemura M., Yoko-o T., Jigami Y. (2006). PER1 Is required for GPI-phospholipase A2 activity and involved in lipid remodeling of GPI-anchored proteins. Mol. Biol. Cell..

[34] Bernat-Silvestre C., Ma Y., Johnson K., Ferrando A., Aniento F., Marcote M.J. (2022). Characterization of *Arabidopsis* post-glycosylphosphatidylinositol attachment to proteins phospholipase 3 like genes. Front. Plant Sci..

[35] Gillmor C.S., Lukowitz W., Brininstool G., Sedbrook J.C., Hamann T., Poindexter P., Somerville C. (2005). Glycosylphosphatidylinositol-anchored proteins are required for cell wall synthesis and morphogenesis in *Arabidopsis*. Plant Cell.

[36] Xu Z., Gao Y., Gao C., Mei J., Wang S., Ma J., Yang H., Cao S., Wang Y., Zhang F. (2022). Glycosylphosphatidylinositol anchor lipid remodeling directs proteins to the plasma membrane and governs cell wall mechanics. Plant Cell.

[37] Vazquez H.M., Vionnet C., Roubaty C., Conzelmann A. (2014). Cdc1 removes the ethanolamine phosphate of the first mannose of GPI anchors and thereby facilitates the integration of GPI proteins into the yeast cell wall. Mol. Biol. Cell.

[38] Yadav P., Yadav S.K., Singh M., Singh M.P., Chinnusamy V. (2024). Genome wide identification and characterization of Isopentenyl transferase (IPT) gene family associated with cytokinin synthesis in rice. Plant Physiol. Rep..

[39] Sun J., Niu Q.-W., Tarkowski P., Zheng B., Tarkowska D., Sandberg G.r., Chua N.-H., Zuo J. (2003). The *Arabidopsis* AtIPT8/PGA22 gene encodes an isopentenyl transferase that Is involved in de novo dytokinin biosynthesis. Plant Physiol..

[40] Sakano Y., Okada Y., Matsunaga A., Suwama T., Kaneko T., Ito K., Noguchi H., Abe I. (2004). Molecular cloning, expression, and characterization of adenylate isopentenyltransferase from hop (*Humulus lupulus* L.). Phytochemistry.

[41] Takei K., Sakakibara H., Sugiyama T. (2001). Identification of genes encoding adenylate isopentenyltransferase, a cytokinin biosynthesis enzyme, in *Arabidopsis thaliana*. J. Biol. Chem..

[42] Wang Y., Shen W., Chan Z., Wu Y. (2015). Endogenous cytokinin overproduction modulates ROS homeostasis and decreases salt stress resistance in *Arabidopsis thaliana*. Front. Plant Sci..

[43] Tsai Y.C., Weir N.R., Hill K., Zhang W., Kim H.J., Shiu S.H., Schaller G.E., Kieber J.J. (2012). Characterization of genes involved in cytokinin signaling and metabolism from rice. Plant Physiol..

[44] Chun Y., Fang J., Savelieva E.M., Lomin S.N., Shang J., Sun Y., Zhao J., Kumar A., Yuan S., Yao X. (2023). The cytokinin receptor OHK4/OsHK4 regulates inflorescence architecture in rice via an IDEAL PLANT ARCHITECTURE1/WEALTHY FARMER’S PANICLE-mediated positive feedback circuit. Plant Cell.

[45] Eichwald T., da Silva L.B., Staats Pires A.C., Niero L., Schnorrenberger E., Filho C.C., Espíndola G., Huang W.L., Guillemin G.J., Abdenur J.E. (2023). Tetrahydrobiopterin: Beyond its traditional role as a cofactor. Antioxidants.

[46] Naponelli V., Noiriel A., Ziemak M.J., Beverley S.M., Lye L.-F., Plume A.M., Botella J.R., Loizeau K., Ravanel S.p., Rébeillé F. (2008). Phylogenomic and runctional analysis of pterin-4a-carbinolamine dehydratase family (COG2154) proteins in plants and microorganisms. Plant Physiol..

[47] Berka V., Yeh H.-C., Gao D., Kiran F., Tsai A.-L. (2004). Redox function of tetrahydrobiopterin and effect of L-arginine on oxygen binding in endothelial nitric oxide synthase. Biochemistry.

[48] Kim H.K., Ha S.H., Han J. (2010). Potential therapeutic applications of tetrahydrobiopterin: From inherited hyperphenylalaninemia to mitochondrial diseases. Ann. N. Y. Acad. Sci..

[49] Peres da Rocha Oliveiros Marciano D., Toledo Ramos F., Neiva Alvim M., Ronaldo Magalhaes J., Giovanni Costa França M. (2010). Nitric oxide reduces the stress effects of aluminum on the process of germination and early root growth of rice. J. Plant Nutr. Soil Sci..

[50] Cao X., Zhu C., Zhong C., Zhang J., Wu L., Jin Q., Ma Q. (2019). Nitric oxide synthase-mediated early nitric oxide burst alleviates water stress-induced oxidative damage in ammonium-supplied rice roots. BMC Plant Biol..

[51] Teng Z., Zheng Q., Peng Y., Li Y., Meng S., Liu B., Peng Y., Duan M., Yuan D., Zhang J. (2025). Nitrate reductase–dependent nitric oxide production mediates nitrate-conferred salt tolerance in rice seedlings. Plant Physiol..

[52] Li Y., Kim J.I., Pysh L., Chapple C. (2015). Four isoforms of *Arabidopsis* 4-coumarate:CoA ligase have overlapping yet distinct roles in phenylpropanoid metabolism. Plant Physiol..

[53] Sun H., Li Y., Feng S., Zou W., Guo K., Fan C., Si S., Peng L. (2013). Analysis of five rice 4-coumarate:coenzyme A ligase enzyme activity and stress response for potential roles in lignin and flavonoid biosynthesis in rice. Biochem. Biophys. Res. Commun..

[54] Li M., Guo L., Wang Y., Li Y., Jiang X., Liu Y., Xie D.-Y., Gao L., Xia T. (2022). Molecular and biochemical characterization of two 4-coumarate: CoA ligase genes in tea plant (*Camellia sinensis*). Plant Mol. Biol..

[55] Singh A., Gupta R., Pandey R. (2016). Rice seed priming with picomolar rutin enhances rhizospheric *Bacillus subtilis* CIM colonization and plant growth. PLoS ONE.

[56] Shomali A., Das S., Arif N., Sarraf M., Zahra N., Yadav V., Aliniaeifard S., Chauhan D.K., Hasanuzzaman M. (2022). Diverse physiological roles of flavonoids in plant environmental stress responses and tolerance. Plants.

[57] Ismail H., Dragišic Maksimovic J., Maksimovic V., Shabala L., Živanovic B.D., Tian Y., Jacobsen S.E., Shabala S. (2015). Rutin, a flavonoid with antioxidant activity, improves plant salinity tolerance by regulating K^+^ retention and Na^+^ exclusion from leaf mesophyll in quinoa and broad beans. Funct. Plant Biol..

[58] Yildiztugay E., Ozfidan-Konakci C. (2015). Profiling of rutin-mediated alleviation of cadmium-induced oxidative stress in *Zygophyllum fabago*. Environ. Toxicol..

[59] Quoc C.D., Lan A.B., Ngoc T.N., Le Hong T., Thanh T.T., Tran G.-B., Hoa S.P., Hung T.N., Ngoc T.N.H., Cong H.N. (2024). Variations in some metabolic compounds in the roots of rice varieties (*Oryza sativa* L.) with different salinity tolerance under salinity stress during the seedling stage. Plant Physiol. Rep..

[60] Tang Y.H., Liu F., Mao K.Q., Xing H.C., Chen J.R., Guo Q.Q. (2018). Cloning and characterization of the key 4-coumarate CoA ligase genes in *Boehmeria nivea*. S. Afr. J. Bot..

[61] Singh A., Gupta R., Pandey R. (2017). Exogenous application of rutin and gallic acid regulate antioxidants and alleviate reactive oxygen generation in *Oryza sativa* L.. Physiol. Mol. Biol. Plants.

[62] Lucas M., Swarup R., Paponov I.A., Swarup K., Casimiro I., Lake D., Peret B., Zappala S., Mairhofer S., Whitworth M. (2010). SHORT-ROOT regulates primary, lateral, and adventitious root development in *Arabidopsis*. Plant Physiol..

[63] Montiel G.g., Gantet P., Jay-Allemand C., Breton C. (2004). Transcription factor networks. pathways to the knowledge of root development. Plant Physiol..

[64] Gallagher K.L., Paquette A.J., Nakajima K., Benfey P.N. (2004). Mechanisms regulating SHORT-ROOT intercellular movement. Curr. Biol..

[65] Cui H., Levesque M.P., Vernoux T., Jung J.W., Paquette A.J., Gallagher K.L., Wang J.Y., Blilou I., Scheres B., Benfey P.N. (2007). An evolutionarily conserved mechanism delimiting SHR movement defines a single layer of endodermis in plants. Science.

[66] Wu S., Lee C.M., Hayashi T., Price S., Divol F., Henry S., Pauluzzi G., Perin C., Gallagher K.L. (2014). A plausible mechanism, based upon Short-Root movement, for regulating the number of cortex cell layers in roots. Proc. Natl. Acad. Sci. USA.

[67] Xing Y., Wang N., Zhang T., Zhang Q., Du D., Chen X., Lu X., Zhang Y., Zhu M., Liu M. (2021). SHORT-ROOT 1 is critical to cell division and tracheary element development in rice roots. Plant J..

[68] Hu M., Pei B.L., Zhang L.F., Li Y.Z. (2014). Histone H2B monoubiquitination is involved in regulating the dynamics of microtubules during the defense response to *Verticillium dahliae* toxins in *Arabidopsis*. Plant Physiol..

[69] Xu Z., Yang Y., Zhang F., Li H., Ma H., Wu W., Ding Y. (2025). OsbZIP27 coordinates with OsHUB1 and OsHUB2 to modulate drought tolerance in rice. J. Genet. Genom..

[70] Xu Z., Li E., Xue G., Zhang C., Yang Y., Ding Y. (2022). OsHUB2 inhibits function of OsTrx1 in heading date in rice. Plant J..

[71] Fleury D., Himanen K., Cnops G., Nelissen H., Boccardi T.M., Maere S., Beemster G.T.S., Neyt P., Anami S., Robles P. (2007). The *Arabidopsis thaliana* homolog of Yeast BRE1 has a function in cell cycle regulation during early leaf and root growth. Plant Cell.

[72] Cao H., Li X., Wang Z., Ding M., Sun Y., Dong F., Chen F., Liu L., Doughty J., Li Y. (2015). Histone H2B monoubiquitination mediated by HISTONE MONOUBIQUITINATION1 and HISTONE MONOUBIQUITINATION2 is involved in anther development by regulating tapetum degradation-related genes in rice. Plant Physiol..

[73] Li Q., Wang L., Grubb L.E., Talasila M., Rodriguez Gallo M.C., Mehta D., Scandola S., Uhrig R.G. (2025). B4 Raf-like MAPKKK RAF24 regulates *Arabidopsis thaliana* flowering time through HISTONE MONO-UBIQUITINATION 2. New Phytol..

[74] Sun M., Shen Y., Chen Y., Wang Y., Cai X., Yang J., Jia B., Dong W., Chen X., Sun X. (2022). Osa-miR1320 targets the ERF transcription factor OsERF096 to regulate cold tolerance via JA-mediated signaling. Plant Physiol..

[75] Dubouzet J.G., Sakuma Y., Ito Y., Kasuga M., Dubouzet E.G., Miura S., Seki M., Shinozaki K., Yamaguchi-Shinozaki K. (2003). OsDREB genes in rice, *Oryza sativa* L., encode transcription activators that function in drought-, high-salt- and cold-responsive gene expression. Plant J..

[76] Wani A.B., Noor W., Pandit A., Husaini A.M. (2025). Upregulated expression of MYB4, DREB1 and AP37 transcription factors modulates cold stress response in high-altitude Himalayan rice via time-dependent ROS regulation. Mol. Biol. Rep..

[77] Xu L., Yang L., Li A., Guo J., Wang H., Qi H., Li M., Yang P., Song S. (2024). An AP2/ERF transcription factor confers chilling tolerance in rice. Sci. Adv..

[78] Cheong Y.H., Moon B.C., Kim J.K., Kim C.Y., Kim M.C., Kim I.H., Park C.Y., Kim J.C., Park B.O., Koo S.C. (2003). BWMK1, a rice mitogen-activated protein kinase, locates in the nucleus and mediates pathogenesis-related gene expression by activation of a transcription factor. Plant Physiol..

[79] Lu J., Ju H., Zhou G., Zhu C., Erb M., Wang X., Wang P., Lou Y. (2011). An EAR-motif-containing ERF transcription factor affects herbivore-induced signaling, defense and resistance in rice. Plant J..

[80] Liu Y., Tian Y., Wang L.-X., Fan T., Zhang J., Chen M.-X., Liu Y.-G. (2021). Phylogeny and conservation of plant U2A/U2A’, a core splicing component in U2 spliceosomal complex. Planta.

[81] Caspary F., Séraphin B. (1998). The yeast U2A′/U2B″ complex is required for pre-spliceosome formation. EMBO J..

[82] Ma L., Zhang X., Li C., Ma X., Zhao X., Zhao X., Zhang P., Zhu X. (2024). A U2 snRNP-specific protein, U2A′, is involved in stress response and drug resistance in Cryptococcus deneoformans. Biochimie.

[83] Bae M.S., Cho E.J., Choi E.-Y., Park O.K. (2003). Analysis of the *Arabidopsis* nuclear proteome and its response to cold stress. Plant J..

[84] Chen L., Weinmeister R., Kralovicova J., Eperon L.P., Vorechovsky I., Hudson A.J., Eperon I.C. (2016). Stoichiometries of U2AF35, U2AF65 and U2 snRNP reveal new early spliceosome assembly pathways. Nucleic Acids Res..

[85] Wang Z., Hong Y., Yao J., Huang H., Qian B., Liu X., Chen Y., Pang J., Zhan X., Zhu J.-K. (2022). Modulation of plant development and chilling stress responses by alternative splicing events under control of the spliceosome protein SmEb in *Arabidopsis*. Plant Cell Environ..

[86] Tang N., Ma S., Zong W., Yang N., Lv Y., Yan C., Guo Z., Li J., Li X., Xiang Y. (2016). MODD mediates deactivation and degradation of OsbZIP46 to negatively regulate ABA signaling and drought resistance in rice. Plant Cell.

[87] Perez J.M., Chen Y., Xiao T.S., Abbott D.W. (2018). Phosphorylation of the E3 ubiquitin protein ligase ITCH diminishes binding to its cognate E2 ubiquitin ligase. J. Biol. Chem..

[88] Kanerva K., Uronen R.-L., Blom T., Li S., Bittman R., Lappalainen P., Peränen J., Raposo G., Ikonen E. (2013). LDL cholesterol recycles to the plasma membrane via a Rab8a-Myosin5b-Actin-Dependent membrane transport route. Dev. Cell.

[89] Sobajima T., Yoshimura S.I., Maeda T., Miyata H., Miyoshi E., Harada A. (2018). The Rab11-binding protein RELCH/KIAA1468 controls intracellular cholesterol distribution. J. Cell Biol..

[90] Kumar K., Gibbs H.C., Yeh A.T., Griffing L.R. (2021). The sterol trafficking pathway in *Arabidopsis thaliana*. Front. Plant Sci..

[91] Joo S.-H., Kim T.-W., Son S.-H., Lee W.S., Yokota T., Kim S.-K. (2011). Biosynthesis of a cholesterol-derived brassinosteroid, 28-norcastasterone, in *Arabidopsis thaliana*. J. Exp. Bot..

[92] Kim B.K., Fujioka S., Takatsuto S., Tsujimoto M., Choe S. (2008). Castasterone is a likely end product of brassinosteroid biosynthetic pathway in rice. Biochem. Biophys. Res. Commun..

[93] Fujii S., Saka H. (2001). The promotive effect of brassinolide on lamina joint-cell elongation, germination and seedling growth under low-temperature stress in rice (*Oryza sativa* L.). Plant Prod. Sci..

[94] Jantapo K., Wimonchaijit W., Wang W., Chaiwanon J. (2021). Supraoptimal brassinosteroid levels inhibit root growth by reducing root meristem and cell elongation in rice. Plants.

[95] Peracchi A., Veiga-da-Cunha M., Kuhara T., Ellens K.W., Paczia N., Stroobant V., Seliga A.K., Marlaire S., Jaisson S., Bommer G.T. (2017). Nit1 is a metabolite repair enzyme that hydrolyzes deaminated glutathione. Proc. Natl. Acad. Sci. USA.

[96] Niehaus T.D., Patterson J.A., Alexander D.C., Folz J.S., Pyc M., MacTavish B.S., Bruner S.D., Mullen R.T., Fiehn O., Hanson A.D. (2019). The metabolite repair enzyme Nit1 is a dual-targeted amidase that disposes of damaged glutathione in *Arabidopsis*. Biochem. J..

[97] Zhang L., Wu M., Teng Y., Jia S., Yu D., Wei T., Chen C., Song W. (2018). Overexpression of the glutathione peroxidase 5 (RcGPX5) gene from *Rhodiola crenulata* increases drought tolerance in *Salvia miltiorrhiza*. Front. Plant Sci..

[98] Song M., Fan X., Chen J., Qu H., Luo L., Xu G. (2020). OsNAR2.1 interaction with OsNIT1 and OsNIT2 functions in root-growth responses to nitrate and ammonium. Plant Physiol..

[99] Forsburg S.L. (2004). Eukaryotic MCM proteins: Beyond replication initiation. Microbiol. Mol. Biol. Rev..

[100] van Deursen F., Sengupta S., De Piccoli G., Sanchez-Diaz A., Labib K. (2012). Mcm10 associates with the loaded DNA helicase at replication origins and defines a novel step in its activation. EMBO J..

[101] Masai H., Taniyama C., Ogino K., Matsui E., Kakusho N., Matsumoto S., Kim J.M., Ishii A., Tanaka T., Kobayashi T. (2006). Phosphorylation of MCM4 by Cdc7 kinase facilitates its interaction with Cdc45 on the chromatin. J. Biol. Chem..

[102] Herridge R.P., Day R.C., Macknight R.C. (2014). The role of the MCM2-7 helicase complex during *Arabidopsis* seed development. Plant Mol. Biol..

[103] Lee Y., Lee D.-E., Lee H.-S., Kim S.-K., Lee W.S., Kim S.-H., Kim M.-W. (2011). Influence of auxins, cytokinins, and nitrogen on production of rutin from callus and adventitious roots of the white mulberry tree (*Morusalba* L.). Plant Cell Tiss. Organ Cult..

[104] Zhang L., Luo P., Bai J., Wu L., Di D.-W., Liu H.-Q., Li J.-J., Liu Y.-L., Khaskheli A.J., Zhao C.-M. (2021). Function of histone H2B monoubiquitination in transcriptional regulation of auxin biosynthesis in *Arabidopsis*. Commun. Biol..

[105] Cai X., Chen Y., Wang Y., Shen Y., Yang J., Jia B., Sun X., Sun M. (2023). A comprehensive investigation of the regulatory roles of OsERF096, an AP2/ERF transcription factor, in rice cold stress response. Plant Cell Rep..

[106] Rebouillat J., Dievart A., Verdeil J.L., Escoute J., Giese G., Breitler J.C., Gantet P., Espeout S., Guiderdoni E., Périn C. (2009). Molecular genetics of rice root development. Rice.

[107] Mao C., He J., Liu L., Deng Q., Yao X., Liu C., Qiao Y., Li P., Ming F. (2020). OsNAC2 integrates auxin and cytokinin pathways to modulate rice root development. Plant Biotechnol. J..

[108] Gao S., Fang J., Xu F., Wang W., Sun X., Chu J., Cai B., Feng Y., Chu C. (2014). CYTOKININ OXIDASE/DEHYDROGENASE4 integrates cytokinin and auxin signaling to control rice crown root formation. Plant Physiol..

[109] Salvi E., Di Mambro R., Pacifici E., Dello Ioio R., Costantino P., Moubayidin L., Sabatini S. (2018). SCARECROW and SHORTROOT control the auxin/cytokinin balance necessary for embryonic stem cell niche specification. Plant Signal Behav..

[110] Md Zali A.Z., Ja’afar Y., Paramisparan K., Ismail S.I., Saad N., Mohd Hata E., Md Hatta M.A., Ismail M.R., Yusof M.T., Zulperi D. (2023). First report of *Burkholderia gladioli* causing bacterial panicle blight of rice in Malaysia. Plant Dis..

[111] Debta H., Kunhamu T.K., Petrík P., Fleischer P., Jisha K.C. (2023). Effect of hydropriming and osmopriming on the germination and seedling vigor of the East Indian Sandalwood (*Santalum album* L.). Forests.

[112] Ros C., Bell R.W., White P.F. (2003). Seedling vigour and the early growth of transplanted rice (*Oryza sativa*). Plant Soil.

[113] Chen Y., Gin J.W., Wang Y., de Raad M., Tan S., Hillson N.J., Northen T.R., Adams P.D., Petzold C.J. (2023). Alkaline-SDS cell lysis of microbes with acetone protein precipitation for proteomic sample preparation in 96-well plate format. PLoS ONE.

[114] Niu L., Zhang H., Wu Z., Wang Y., Liu H., Wu X., Wang W. (2018). Modified TCA/acetone precipitation of plant proteins for proteomic analysis. PLoS ONE.

[115] Waterborg J.H., Matthews H.R. (1994). Basic Protein and Peptide Protocols.

[116] Tyanova S., Temu T., Cox J. (2016). The MaxQuant computational platform for mass spectrometry-based shotgun proteomics. Nat. Protoc..

[117] Pang Z., Lu Y., Zhou G., Hui F., Xu L., Viau C., Spigelman A.F., MacDonald P.E., Wishart D.S., Li S. (2024). MetaboAnalyst 6.0: Towards a unified platform for metabolomics data processing, analysis and interpretation. Nucleic Acids Res..

[118] Blom N., Sicheritz-Pontén T., Gupta R., Gammeltoft S., Brunak S. (2004). Prediction of post-translational glycosylation and phosphorylation of proteins from the amino acid sequence. PROTEOMICS.

[119] Kolberg L., Raudvere U., Kuzmin I., Adler P., Vilo J., Peterson H. (2023). g:Profiler—Interoperable web service for functional enrichment analysis and gene identifier mapping (2023 update). Nucleic Acids Res..

[120] Ødum M.T., Teufel F., Thumuluri V., Almagro Armenteros J.J., Johansen A.R., Winther O., Nielsen H. (2024). DeepLoc 2.1: Multi-label membrane protein type prediction using protein language models. Nucleic Acids Res..

[121] Szklarczyk D., Santos A., von Mering C., Jensen L.J., Bork P., Kuhn M. (2016). STITCH 5: Augmenting protein-chemical interaction networks with tissue and affinity data. Nucleic Acids Res..

[122] R Core Team (2013). R: A Language and Environment for Statistical Computing.

[123] Villanueva R.A.M., Chen Z.J. (2019). ggplot2: Elegant graphics for data analysis. Meas. Interdiscip. Res. Perspect..

