# Supplementary material for: Phosphoproteomic Analysis Reveals Impairment of Rice Germination by Chloramphenicol
Source: Plants (Basel). 2025 Sep 12;14(18):2845. doi: 10.3390/plants14182845 (PMC12473894; doi:10.3390/plants14182845)
Supplement: Supplementary file 1 [file plants-14-02845-s001.zip › Supplementary Figures.pdf]

# Supplementary Figures

## Phosphoproteomic Analysis Reveals Impairment of Rice Germination by Chloramphenicol

**Rui Li[1,2], Narumon Phaonakrop[3], Sittiruk Roytrakul[3], Karan Lohmaneeratana[2], Arinthip Thamchaipenet[2,4,*]**

1 Interdisciplinary Graduate Program in Bioscience, Faculty of Science, Kasetsart University, Bangkok 10900, Thailand; rui.l@ku.th (R.L.);
2 Department of Genetics, Faculty of Science, Kasetsart University, Bangkok 10900, Thailand; rui.l@ku.th (R.L.); karan.l@ku.th (K.L.); arinthip.t@ku.ac.th (A.T.);
3 National Center for Genetic Engineering and Biotechnology, National Science and Technology Development Agency, Pathum Thani 12120, Thailand; narumon.pha@biotec.or.th (N.P.); sittiruk@biotec.or.th (S.R.)
4 Omics Center for Agriculture, Bioresources, Food, and Health, Kasetsart University (OmiKU), Bangkok 10900, Thailand; arinthip.t@ku.ac.th (A.T)

* Correspondence: arinthip.t@ku.ac.th;

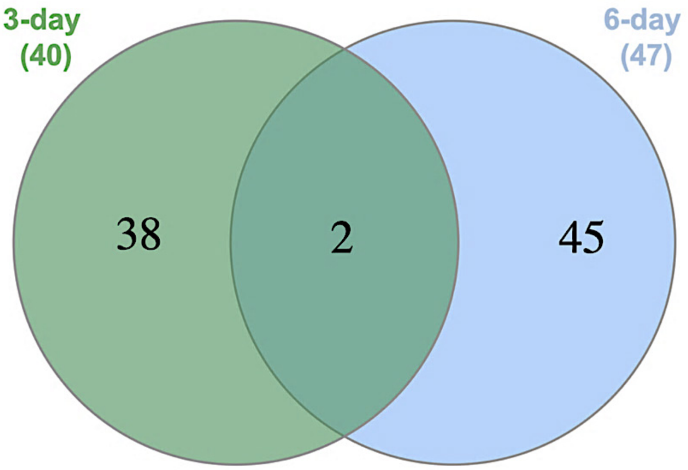


**Figure S1.** Venn diagram of differentially expressed phosphoproteins (DEPPs) identified at 3-day (germination stage) and 6-day (early seedling establishment stage) in response to chloramphenicol (CAM).

**tr_Q2QQ28_Q2QQ28_ORYSJ**

**Predicted localizations:** Nucleus

**Predicted membrane association:** Soluble

**Predicted signals:** Nuclear localization signal

| Localization | Cytoplasm | Nucleus | Extracellular | Cell membrane | Mitochondrion | Plastid | Endoplasmic reticulum | Lysosome/Vacuole | Golgi apparatus | Peroxisome |
|---|---|---|---|---|---|---|---|---|---|---|
| Probability | 0.4554 | 0.6476 | 0.0942 | 0.1379 | 0.0957 | 0.0299 | 0.1270 | 0.0725 | 0.0921 | 0.0106 |

| Membrane association | Peripheral | Transmembrane | Lipid anchor | Soluble |
|---|---|---|---|---|
| Probability | 0.3090 | 0.1200 | 0.0960 | 0.7830 |

**Figure S2.** Subcellular localization prediction of the CCHC-type zinc finger protein (Q2QQ28), generated using DeepLoc version 2.1.

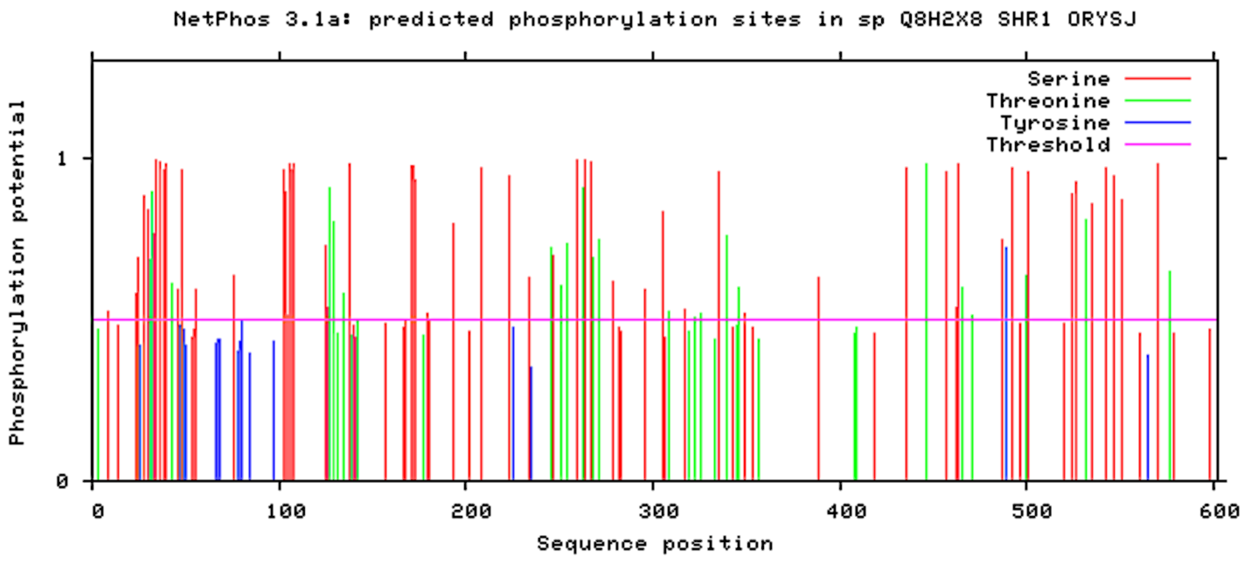


**Figure S3.** Predicted phosphorylation sites of OsSHR1 (Q8H2X8), generated using NetPhos version 3.1.
